# The role of gender in health insurance enrollment among geriatric caregivers: results from the 2022 informal caregiving, health, and healthcare survey in Ghana

**DOI:** 10.1186/s12889-024-18930-y

**Published:** 2024-06-11

**Authors:** Williams Agyemang-Duah, Michael Safo Oduro, Prince Peprah, Dina Adei, Jacob Oppong Nkansah

**Affiliations:** 1 Department of Geography and Planning, Queen’s University, Kingston, ON K7L 3N6 Canada; 2 Pfizer Research and Development, PSSM Data Sciences, Groton, CT 06340 USA; 3 Social Policy Research Centre, University of New South Wales, Sydney, Australia; 4 Centre for Primary Health Care and Equity, University of New South Wales, Sydney, Australia; 5 Department of Planning, Kwame Nkrumah University of Science and Technology, Private Mail Bag, Kumasi, Ghana; 6 School of Graduate Studies, Lingnan University, Tuen Mun, Hong Kong

**Keywords:** Gender, Health insurance enrollment, Geriatric informal caregivers, Ghana

## Abstract

**Background:**

Female informal caregivers of older adults experience a higher burden of physical and mental health problems compared to their male counterparts due to the greater intensity of care they provide. This is likely to result in an imbalance in health needs, including health insurance enrollment, between male and female informal caregivers of older adults. However, to date, no study is available on the role of gender in health insurance enrollment among informal caregivers of older adults in Ghana. This study examines the association between gender and health insurance enrollment among informal caregivers of older adults in Ghana.

**Methods:**

Cross-sectional data from the Informal Caregiving, Health, and Healthcare Survey among caregivers of older adults aged 50 years or above (*N* = 1,853 and mean ages =   39.15 years and  75.08 years of informal caregivers and their care recipients, respectively) in Ghana were analyzed. A binary logit regression model was used to estimate the association between gender and health insurance enrollment. All statistical inferences were made at the 5% significance level.

**Results:**

The final Model (3) showed that female informal caregivers were 2.70 times significantly more likely to enrol in a health insurance scheme than their male counterparts (AOR: 2.70, 95% CI: 2.09–3.48, *p*-value = 0.001). Apart from gender, the results revealed that participants aged 55–64 years (AOR = 2.38, 95%CI: 1.29–4.41, *p*-value = 0.006), with tertiary education (AOR: 3.62, 95% CI: 2.32–5.66, *p*-value = 0.001) and living with the care recipients (AOR: 1.50, 95% CI: 1.14–1.98, *p*-value = 0.003) were significantly more likely to enrol in a health insurance scheme than their counterparts. The findings further showed that those who earned between GH¢1000 and 1999 (US$99.50-198.50) monthly (AOR: 0.70, 95% CI: 0.52–0.95, *p*-value = 0.022) and were affiliated with African traditional religion (AOR: 0.30, 95%CI: 0.09–0.99, *p*-value = 0.048) were significantly less likely to enrol in a health insurance scheme than their counterparts.

**Conclusion:**

Gender was a significant predictor of health insurance enrollment among informal caregivers of older adults. This finding contributes to the empirical debates on the role of gender in health insurance enrollment among informal caregivers of older adults. Policymakers need to develop gender-specific measures to address gender gaps in health insurance enrollment among informal caregivers of older adults in Ghana. Such health policies and programs should consider other significant demographic and socioeconomic factors associated with health insurance enrolment among informal caregivers of older adults in Ghana.

## Introduction

Universal health coverage (UHC) remains a global health priority and is one of the key targets of the Sustainable Development Goals (SDGs) [1]. For instance, target 3.8 of the SDGs seeks to achieve UHC, including access to quality essential healthcare services and safe, effective, quality, and affordable necessary medicines and vaccines for all [1]. One of the prominent approaches to achieving UHC is implementing universal/national health insurance schemes, especially in developing countries, including Ghana, where access to essential healthcare services is predominantly hindered by financial barriers [2, 3]. Ghana has been a pioneer in ensuring UHC in sub-Saharan Africa (SSA), setting the standard for the health insurance model to undo financial challenges associated with healthcare utilization [4, 5]. Thus, between 2017 and 2021, the percentage of active enrollees in the National Health Insurance Scheme (NHIS) increased from 10.6 million to 16.8 million (57.3%), including persons aged 18 (41.6%), informal sector workers (36.4%) and older adults aged 70 years or above (less than 5%) [6].

Previous studies suggest gender gaps in health insurance enrollment among the general population. For instance, Ayanore et al. [7] drawing data from the 2014 Ghana Demographic Health Survey, found that 66% of females and 52.6% of males are enrolled in the NHIS. Further, Salari et al. [8] based on the Ghana Living Standards Survey (GLSS 2012–2013) reported that 38.1% of females and 34.4% of males have a valid health insurance card. These reports suggest that females have greater enrollment rates in NHIS than their male counterparts [9–12]. The literature suggests that males are more risk-averse and display apathetic behaviours in health-related issues [13, 14]. Yet, scholars such as Dixon et al. [15] and Alatinga and Williams [16] have argued that male-headed families were more likely to sign up for health insurance schemes than female-headed households, with the feminization of poverty as the underlying cause.

Why geriatric caregivers? Several arguments can be advanced in this regard. For instance, one plausible reason is the increase in the population of older adults globally [17] and the need to meet their varied care needs [18]. In Ghana, like in many other countries, the caregiving role has historically been seen as the responsibility of females, especially when the care receivers are family members such as parents, spouses, and siblings [19]. However, lately, male involvement in informal caregiving is increasing [19, 20]. During care provision, caregivers suffer from varied comorbidities, such as physical and mental health issues, which may result from the provision of care [21, 22]. Due to their greater intensity of providing informal care, female informal caregivers of older adults are likely to experience a higher burden of physical and mental health problems compared to their male counterparts. This is likely to result in an imbalance of health insurance enrollment between male and female informal caregivers of older adults. However, to the best of our knowledge, there is no study on the role of gender in health insurance enrollment among informal caregivers of older adults in SSA, including Ghana.

Understanding the role of gender in health insurance enrollment among informal caregivers of older adults may help inform gender-specific policies and programs to bolster health insurance enrollment among this population group. This study examines the role of gender in health insurance enrollment among informal caregivers of older adults in Ghana. In this study, we hypothesize that female informal caregivers of older adults are significantly more likely to enrol in a health insurance scheme in Ghana.

## Materials and methods

### Study design and sampling procedure

In this study, we obtained data from a large cross-sectional survey on informal caregiving, health, and healthcare among caregivers of older adults 50 years or above conducted between July and September 2022.

This study was conducted in the Ashanti Region of Ghana, which has 43 Metropolitan, Municipal and Districts (hereafter, districts). We followed more rigorous scientific procedures to select a representative sample of districts for the study. First, we used cluster sampling to split the study area into three geographical zones: northern, middle, and southern. This was to ensure the representativeness and generalization of the study findings. The demarcation of the study area was guided by the geographical location, socio-economic status, and cultural disparities [19]. Each district in the study area was tied to one of the three categories. Second, we applied a simple random sampling technique to select a specific number of districts from each demarcated zone. This was to give every district in the study area an equal chance of being selected. That is, three districts were selected from the northern zone (Offinso Municipal, Ejura-Sekyedumase Municipal and Sekyere Central District), three were chosen from the southern zone (Adansi-South District, Bekwai Municipal and Obuasi Municipal), and seven were picked from the middle zone (Kumasi Metropolis, Atwima Nwabiagya Municipal, Sekyere-Kumawu District, Ejisu Municipal, Kwadaso Municipal, Asokwa Municipal and Oforikrom Municipal). More districts were selected from the middle zone because most of the districts in the study area are clustered in the middle zone. Detailed information on how simple random sampling was applied to select the study districts and the number of communities included in this study has been reported in a previous study [19].

A snowball sampling technique was employed to recruit the participants for the study. This sampling approach was adopted for the following reasons: first, we did not have data on the number of caregivers who provide informal care for older adults in the study area and; second, we did not know any caregiver(s) in the study area who provided informal care for older adults. We applied the snowball sampling technique as follows. First, we started by contacting community stakeholders in the study area and letting them know the purpose of the study. Since they were natives of the communities, they could identify participants who cared for older adults in the study area. When we finished interviewing participants, we asked if they knew anyone who provided informal care for older adults. Thus, current participants were able to recommend potential participants who also provide informal care for older adults to participate in the survey.

The sample included those aged 18 years or above and providing informal care for an older adult aged 50 years or above. Detailed information about the inclusion and exclusion criteria can be found elsewhere [19].

**Fig. 1:**
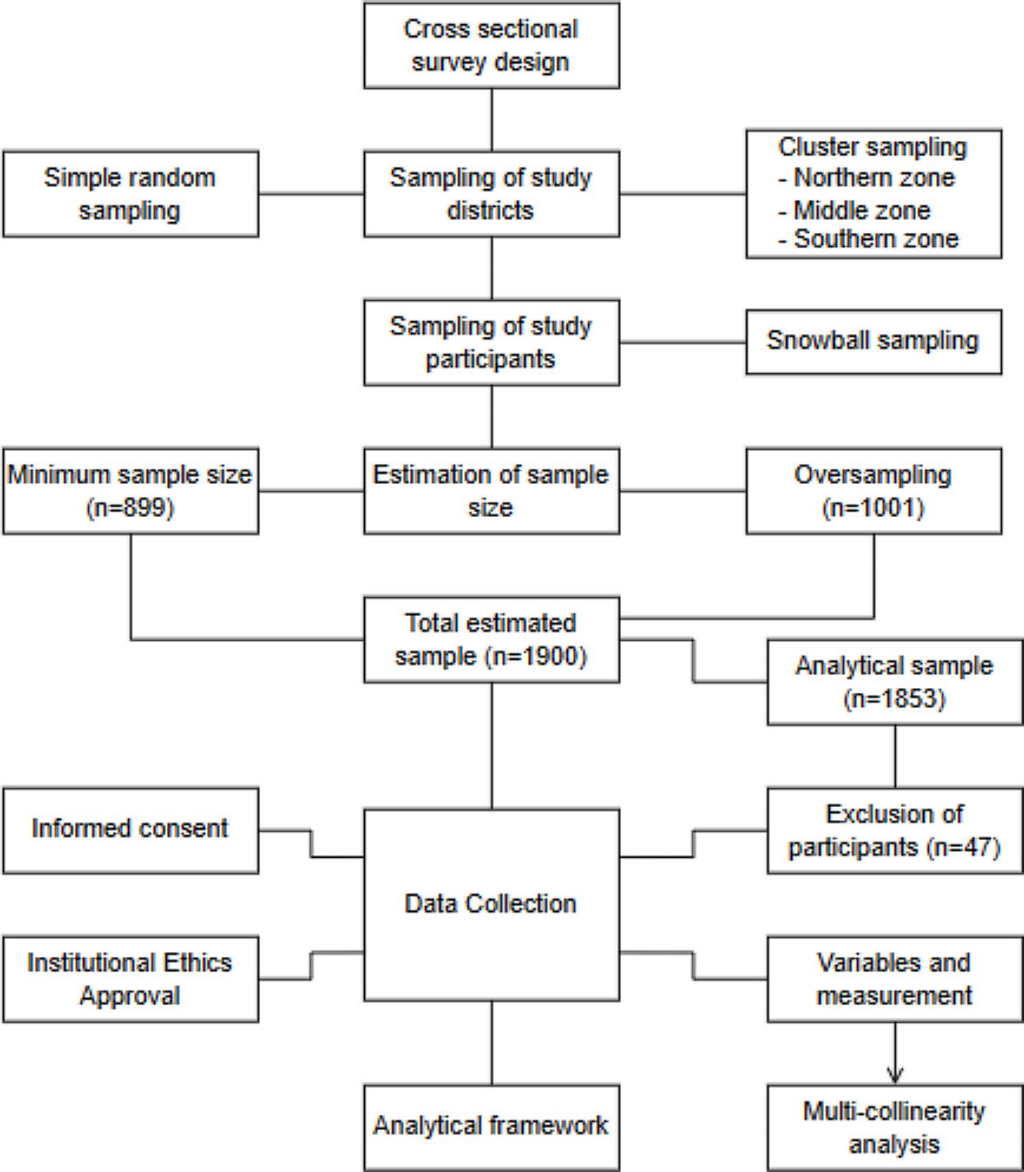
Flow chart showing an overview of the study’s methods

Following the appropriate formula and procedures for sample size estimation, which has been comprehensively outlined in an earlier study [19], our estimated sample size for this study was 1,900 informal caregivers of older adults (see Fig. 1). However, the analytic sample was restricted to 1,853 informal caregivers of older adults due to the following circumstances. First, 36 participants, representing 1.89%, declined to participate in the study. Second, 7 participants, constituting 0.37%, provided incomplete responses. Last, 4 participants, representing 0.21% of the participants’ responses, included missing data. Consequently, the response rate was 97.52% (*n* = 1,853) [19].

### Data collection procedure

An interviewer-administered questionnaire was employed to collect data. The questionnaire covered questions on demographic, socio-economic, health-related characteristics and health insurance status. We entered all the questions in the questionnaire into Qualtrics, an electronic survey tool, to capture participants’ responses digitally. We drafted the questionnaire in English, which was read in Twi (the local language in the study area) during the fieldwork. Lasting between 30 and 35 min, the data collection took place in the participants’ homes and was free from the interference of any third party. Detailed information on the data collection procedure has been reported in a previous study [19].

### Ethics approval

Approval for the study site was obtained from the Ashanti Regional Health Directorate under the Ghana Health Service (Ref: GHS/ASH/RES/V.2). Ethical approvals were obtained from the General Research Ethics Board (GREB), Queen’s University, Kingston, Canada (Ref: GGEOPL-344-22) and the Committee on Human Research Publication and Ethics (CHRPE), School of Medical Sciences, College of Health Sciences, KNUST, Kumasi, Ghana (Ref: CHRPE/AP/182/22). Informed consent, both verbal and written, was obtained from the participants. In the case of non-literate participants, we obtained consent from their legal guardians, which the ethics committee sanctioned.

### Measures

#### Dependent variable

Health insurance enrollment was considered the dependent variable in this study. This variable was binary and was premised on a survey question interrogating informal caregivers of older adults: “Do you have insurance that covers your healthcare expenditure?”. This variable was coded “0” for those who responded “No” and “1” for those who responded “Yes”. Measuring health insurance enrollment using a dichotomous variable is consistent with previous studies in Ghana, which focused on the general population, informal sector workers, persons with disabilities and older adults [12, 23–25].

#### Independent and control variables

The primary independent variable was gender, measured as male = 0 and female = 1. Other control variables were age (years) (0 = 18–24, 1 = 25–34, 2 = 35–44, 3 = 45–54, 4 = 55–64, and 5 = 65 or above), marital status (0 = never married, 1 = currently married, 2 = separated/widowed/ divorced), place of residence (0 = rural, 1 = urban), ethnicity (0 = Akan, 1 = non-Akan), religious affiliation (0 = Christianity, 1 = Islam, 2 = African traditional religion, 3 = no religion), education level (0 = no formal education, 1 = primary, 2 = junior high school, 3 = senior high school, 4 = tertiary), employment status (0 = employed, 1 = unemployed), living with the care recipient (0 = no, 1 = yes), income level (GH¢) (0 = less than 1000, 1 = 1000–1999, 2 = 2000 or above) and the self-rated health status (0 = very poor/poor, 1 = fair, 2 = good, 3 = very good, 4 = excellent) of caregivers. To enhance the robustness of our results, we checked for multicollinearity for all our independent and control variables. The variance inflation factor (VIF) for all the independent and control variables was less than 2. For instance, the minimum VIF was 1.05, and the maximum VIF was 1.58. These figures demonstrate no strong multicollinearity among this study’s independent and control variables.

### Analytical framework

The objective of this study was addressed statistically via descriptive and inferential methods. First, the data were explored to gain insight and examine probable trends in background characteristics and the prevalence of health insurance enrollment among the participants. Specifically, descriptive statistics regarding counts and proportions were obtained on the dependent variable, and demographic, socioeconomic and health predictors were considered. A contingency table is further presented to summarize the relationships between health insurance enrollment and the predictor variables. Additionally, a variable importance metric was established based on a Random Forest Approach [26] to ascertain which predictor variables mostly contributed to health insurance enrollment among the participants. Subsequently, three binary logit models were fitted sequentially. The first Model only considered gender as the primary predictor variable. The second multivariable binary logit model estimated the added effect of other demographic and socio-economic variables in predicting enrollment in health insurance schemes. The final Model comprised all considered predictor variables. Generally, for a set of explanatory variables $\varvec{X}=\left\{{x}_{0},{x}_{1},\dots ,{x}_{i}\right\},$ with coefficients $\varvec{\vartheta }= \left\{{\vartheta }_{0},{\vartheta }_{1},...,{\vartheta }_{i}\right\},$the binary logit model was specified as,

$${Y}_{i}| {\vartheta }_{0},{\vartheta }_{1},...,{\vartheta }_{i}\sim\text{B}\text{e}\text{r}\text{n}\text{o}\text{u}\text{l}\text{l}\text{i}({\pi }_{r})$$

$${\rm{with,}}\,{\rm{log}}\left( {\frac{{{\pi _i}}}{{1 - {\pi _i}}}} \right) = {X^\prime }\vartheta$$

Where ${\pi }_{i}$ represents the probability of the dependent measure, ${Y}_{i}$ is modelled, and $\text{l}\text{o}\text{g}\left(\frac{{\pi }_{i}}{1-{\pi }_{i}}\right)$represents the log odds of the probabilities. Based on this Model, the adjusted odds ratio of parameter estimates, 95% confidence intervals and *p*-values are obtained. All statistical analyses were implemented in R software, and inferences were made at a 5% significance level.

## Results

### Sample characteristics

Table 2 highlights the sample characteristics of the participants. The results showed that 27.7% of the participants were aged 25–34 years. The mean age of the participants was 39.15 years, and a standard deviation of 13.28 years. It was further observed that 72.9% of the participants self-identified as female, 24% had a junior or senior high school education, 80.7% were Christians, and 33.6% were unemployed. A considerable proportion (76.80%) had an income of less than GH¢1000 [US$99.50 as at the time of the field survey, September 2022) (mean income = GH¢683.24, standard deviation =GH¢ 835.64), and 56.7% were urban residents. Furthermore, 79.6% were living with their care recipients, 76.2% were Akan, and 27.7% rated their health as excellent. Details of the results are shown in Tables 1 and 2.

**Table 1:** Contingency table of health insurance enrollment and covariates

Variables	Levels/Categories	Health Insurance Enrollment	
		No Proportion (sample)	Yes Proportion (Sample)
Age (years) of caregivers	18–24	26.69% (71)	73.31% (195)
	25–34	24.95% (128)	75.05% (385)
	35–44	22.55% (99)	77.45% (340)
	45–54	24.39% (90)	75.61% (279)
	55–64	15.12% (26)	84.88% (146)
	65 or above	21.28% (20)	78.72% (74)
Gender of caregivers	Male	35.66% (179)	64.34% (323)
	Female	18.87% (255)	81.13% (1096)
Residence of caregivers	Rural	25.56% (205)	74.44% (597)
	Urban	21.79% (229)	78.21% (822)
Ethnicity of caregivers	Akan	22.80% (322)	77.20% (1090)
	Non-Akan	25.40% (112)	74.60% (329)
Religious affiliation of caregivers	Christianity	22.47% (336)	77.53% (1159)
	Islam	25.76% (76)	74.24% (219)
	African Traditional Religion	61.54% (8)	38.46% (5)
	Other	28.00% (14)	72.00% (36)
Education level of caregivers	No formal education	28.49% (151)	71.51% (379)
	Primary	17.76% (27)	82.24% (125)
	Junior High School	22.92% (102)	77.08% (343)
	Senior high school	25.17% (112)	74.83% (333)
	Tertiary	14.95% (42)	85.05% (239)
Employment status of caregivers	Unemployed	26.53% (165)	73.47% (457)
	Employed	21.85% (269)	78.15% (962)
Marital status of caregivers	Never married	27.09% (152)	72.91% (409)
	Currently Married	21.57% (223)	78.43% (811)
	Separated/Widowed/ Divorced	22.87% (59)	77.13% (199)
Living with the care recipient of caregivers	No	29.89% (113)	70.11% (265)
	Yes	21.76% (321)	78.24% (1154)
Income (GH¢) of caregivers	Less than 1000	22.36% (318)	77.64% (1104)
	1000–1999	27.42% (82)	72.58% (217)
	2000 or above	25.76% (34)	74.24% (98)
Self-rated health status of caregivers	Very poor/poor	21.43% (6)	78.57% (22)
	Fair	34.72% (25)	65.28% (47)
	Good	24.65% (87)	75.35% (266)
	Very good	25.03% (222)	74.97% (665)
	Excellent	18.32% (94)	81.68% (419)

**Table 2:** Descriptive statistics of the participants

Descriptive statistics of the participants						
Variables	**Levels/Categories**	**Proportion (Sample)**	**Mean**	**Median**	**Mode**	**Std. Deviation**
Age (years) of caregivers	18–24	14.40% (266)	39.15	38	45	13.28
	25–34	27.70% (513)				
	35–44	23.70% (439)				
	45–54	19.90% (369)				
	55–64	9.30% (172)				
	65 or above	5.10% (94)				
Gender of caregivers	Male	27.10% ( 502)				
	Female	72.90% (1351)				
Place of residence of caregivers	Rural	43.30% (802)				
	Urban	56.70% (1051)				
Ethnicity of caregivers	Akan	76.20% (1412)				
	Non-Akan	23.80% (441)				
Religious affiliation of caregivers	Christianity	80.70% (1495)				
	Islam	15.90% (295)				
	African Traditional Religion	0.70% (13)				
	Other	2.70% (50)				
Education level of caregivers	No formal education	28.60% (530)				
	Primary	8.20% (152)				
	Junior High School	24% (445)				
	Senior high school	24% (445)				
	Tertiary	15.20% (281)				
Employment status of caregivers	Unemployed	33.60% (622)				
	Employed	66.40%(1231)				
Marital status of caregivers	Never married	30.30% (561)				
	Currently Married	55.80% (1034)				
	Separated/Widowed/ Divorced	13.90% (258)				
Living with the care recipient	No	20.40% (378)				
	Yes	79.60% (1475)				
Income of caregivers (GH¢)	Less than 1000	76.80% (1422)	683.24	500	500	835.64
	1000–1999	16.10% (299)				
	2000 or above	7.10% (132)				
Self-rated health status of caregiver	Very poor/poor	1.50% (28)				
	Fair	3.90% (72)				
	Good	19.10% (353)				
	Very good	47.90% (887)				
	Excellent	27.70% (513)				

### Health insurance enrollment rate among the participants by gender

**Fig. 2:**
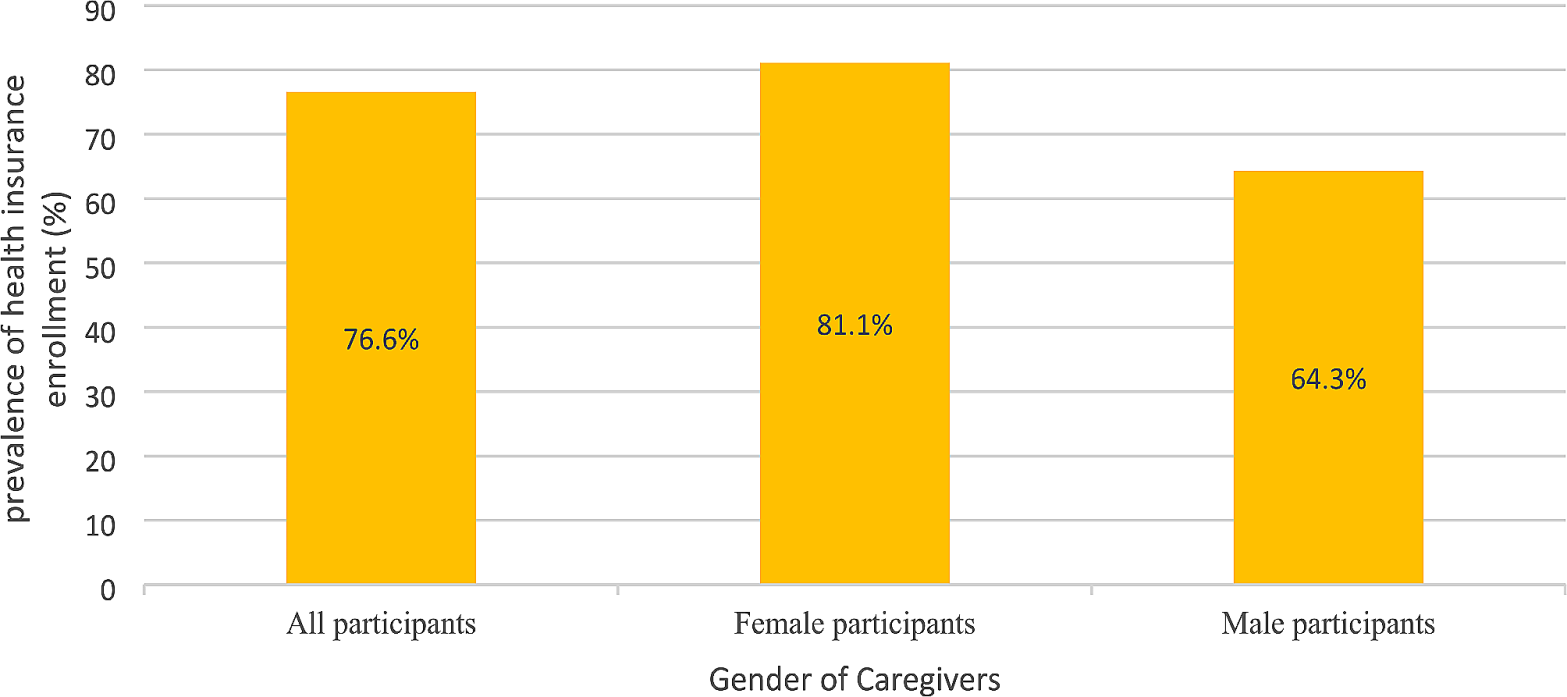
Prevalence of enrollment in a health insurance scheme by gender

As reported in Fig. 2, 76.6% of the participants were enrolled in a health insurance scheme. The results showed that 81.1% of female participants were enrolled in a health insurance scheme. Also, 64.3% of the male participants were enrolled in a health insurance scheme (see Fig. 2).

### Mean decrease Gini metric

Mean Decrease Gini values were estimated from a Random Forest analysis to ascertain which predictor variables most likely contribute to health insurance enrollment. They were used to curate a variable importance measure. The Mean Decrease Gini is a metric commonly used in decision tree-based machine learning algorithms, particularly in Random Forests [26, 27]. It assesses the importance of different variables in making accurate predictions. The variable importance measure aggregates the Mean Decrease Gini values across all trees in the Random Forest. This provides a comprehensive measure of the overall impact of each variable on the Model’s performance and provides a holistic assessment of each variable’s importance in the Model. This information is valuable for identifying key contributing factors to health insurance enrollment and improving the Model’s interpretability and efficiency.

**Fig. 3:**
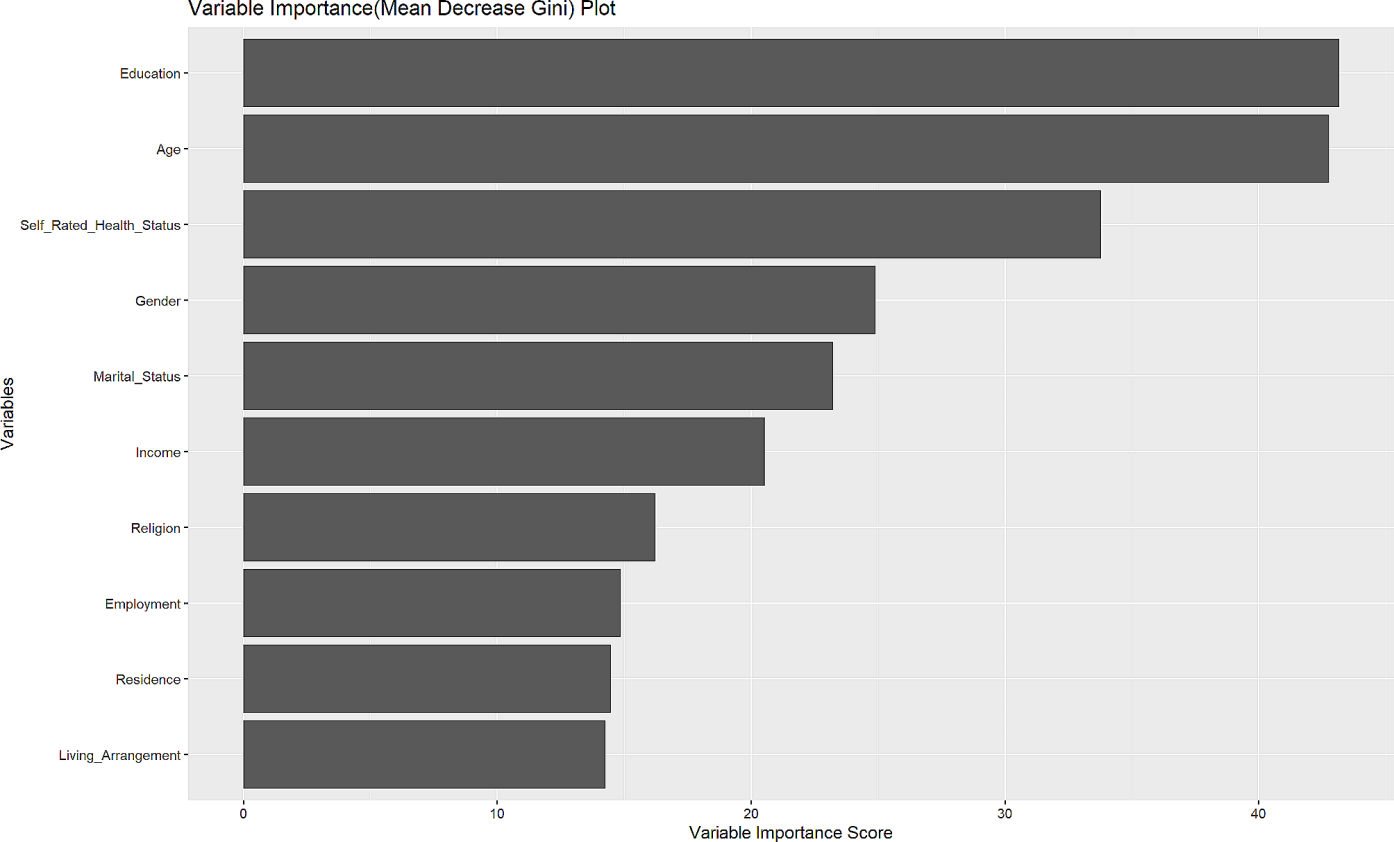
Random forests variable importance plot

Table 3; Fig. 3 represent Mean Decrease Gini values and associated variable importance plots, respectively. The higher the Mean Decrease Gini, the more critical the factor is in predicting health insurance enrollment. In line with this explanation, the results broadly indicate that level of education, age, self-rated health status and the gender of informal caregivers of older adults were potentially the most contributing factors to health insurance enrollment, given their dominant mean decrease Gini values. This is also evidenced in Fig. 3.

**Table 3:** Variable importance results via the mean decrease Gini metric

Variables	Mean Decrease Gini
Age (years) of caregivers	**42.996**
Gender of caregivers	**25.155**
Residence of caregivers	**15.098**
Ethnicity of caregivers	**13.442**
Religious affiliation of caregivers	**15.924**
Education level of caregivers	**43.600**
Employment status of caregivers	**14.853**
Marital status of caregivers	**22.636**
Living arrangements with the care recipient	**14.010**
Income of caregivers (GH¢)	**20.452**
Self-rated health status of caregiver	**33.951**

### Association between gender and health insurance enrollment among informal caregivers of older adults

Table 4 provides information on the association between gender and health insurance enrollment among informal caregivers of older adults. In Model 1, the results showed that females were 2.38 times significantly more likely to enrol in a health insurance scheme than their male counterparts (COR = 2.38, 95%CI: 1.90–2.99, *p*-value = 0.001). In Model 2, when other socio-economic and demographic variables were added, the association between gender and health insurance enrollment persisted. For instance, the findings indicated that females were 2.79 times significantly more likely to enrol in a health insurance scheme than males (AOR = 2.79, 95%CI: 2.170–3.59, *p*-value = 0.001). In Model 2, we observed changes in the odds of health insurance enrollment between male and female participants when we added demographic and socio-economic variables to the variable in Model (1). For instance, it was observed that the likelihood of female participants enrolling in a health insurance scheme increased from 2.38 in Model 1 to 2.79 in Model (2). This suggests a 41% increased in the likelihood of health insurance enrollment among female participants when demographic and socio-economic variables were included in Model 2. This change suggests that demographic and socio-economic variables widen the gender gaps in health insurance enrollment among the participants.

**Table 4:** Association between gender and health insurance enrollment

Model 1: Multiple Binary Logistic Regression Results with Gender as Covariate				
Variable	**COR**	**P-value**	**OR2.5%**	**OR97.5%**
(Intercept)	1.800	0.001	1.500	2.160
Gender of caregivers (Ref = Male)				
Female	2.38	0.001	1.900	2.990
Model 2: Multiple Binary Logistic Regression Results with Gender and Other Demographic and Socio Economic Variables				
Variable	**AOR**	**P-value**	**OR2.5%**	**OR97.5%**
(Intercept)	0.442	0.001	0.262	0.743
Gender of caregivers (Ref = Male)				
Female	2.790	0.001	2.170	3.590
Age (years) of caregivers (Ref = 18–24)				
25–34	1.006	0.975	0.688	1.471
35–44	1.200	0.438	0.760	1.900
45–54	1.170	0.524	0.720	1.910
55–64	2.160	0.013	1.170	3.970
65 or above	1.640	0.152	0.830	3.210
Place of residence of caregivers (Ref = Rural)				
Urban	1.100	0.404	0.880	1.390
Ethnicity of caregivers (Ref = Akan)				
Non-Akan	0.960	0.820	0.660	1.390
Religious affiliation of caregivers (Ref = Christianity)				
Islam	1.150	0.519	0.750	1.780
African Traditional Religion	0.300	0.049	0.090	1.000
Other	1.150	0.675	0.590	2.250
Education level of caregivers (Ref = No formal education)				
Primary	2.080	0.003	1.290	3.360
Junior High School	1.590	0.005	1.150	2.200
Senior high school	1.700	0.002	1.210	2.400
Tertiary	3.720	0.001	2.390	5.800
Employment status of caregivers (Ref = Unemployed)				
Employed	1.260	0.086	0.970	1.640
Marital status of caregivers (Ref = Never married)				
Currently Married	1.360	0.067	0.980	1.880
Separated/Widowed/ Divorced	1.140	0.539	0.740	1.760
Living with the care recipient (Ref = No)				
Yes	1.570	0.001	1.200	2.060
Income of caregivers (GH¢) (Ref = Less than 1000)				
1000–1999	0.700	0.022	0.520	0.950
2000 or above	0.780	0.254	0.500	1.200
Model 3: Multiple Binary Logistic Regression for all variables				
Variable	**AOR**	**P-value**	**OR2.5%**	**OR97.5%**
(Intercept)	0.579	0.32578	0.203	1.841
Gender of caregivers (Ref = Male)				
Female	2.700	0.001	2.090	3.480
Age (years) of caregivers (Ref = 18–24)				
25–34	1.060	0.78	0.720	1.550
35–44	1.260	0.321	0.800	2.000
45–54	1.250	0.365	0.770	2.050
55–64	2.380	0.006	1.290	4.410
65 or above	1.820	0.087	0.920	3.590
Residence of caregivers (Ref = Rural)				
Urban	1.140	0.279	0.900	1.440
Ethnicity of caregivers (Ref = Akan)				
Non-Akan	0.960	0.831	0.660	1.400
Religious affiliation of caregivers (Ref = Christianity)				
Islam	1.150	0.526	0.740	1.780
African Traditional Religion	0.300	0.048	0.090	0.990
No religion	1.300	0.450	0.660	2.550
Education level of caregivers (Ref = No formal education)				
Primary	2.080	0.003	1.280	3.360
Junior High School	1.570	0.006	1.140	2.180
Senior high school	1.670	0.004	1.180	2.360
Tertiary	3.620	0.001	2.320	5.660
Employment status of caregivers (Ref = Unemployed)				
Employed	1.240	0.106	0.950	1.620
Marital status of caregivers (Ref = Never married)				
Currently Married	1.350	0.069	0.980	1.880
Separated/Widowed/ Divorced	1.160	0.506	0.750	1.790
Living with the care recipient (Ref = No)				
Yes	1.500	0.003	1.140	1.980
Income of caregivers (GH¢) (Ref = Less than 1000)				
1000–1999	0.700	0.022	0.520	0.950
2000 or above	0.780	0.259	0.500	1.200
Self-rated health status of caregivers (Ref = Very poor/poor)				
Fair	0.490	0.195	0.16	1.45
Good	0.690	0.458	0.25	1.86
Very good	0.720	0.515	0.27	1.92
Excellent	0.990	0.979	0.36	2.68

In the final Model (3), when the health-related variable was added to the socioeconomic and demographic variables, the association between gender and health insurance enrollment was still present. The results in Model 3 showed that females were 2.70 times more likely to enrol in a health insurance scheme than their male counterparts (AOR: 2.70, 95%CI: 2.09–3.48, *p*-value = 0.001). In Model 3, we observed that when a health-related variable was added to all variables in Model 2, we observed a decrease in the likelihood of health insurance enrollment among female participants by 9%. That is from 2.79 in Model 2 to 2.70 in Model 3. This suggests that when we added health-related variables to demographic and economic variables in Model 3, the health-related variable was able to close the gaps between male and female participants regarding health insurance enrollment slightly.

Aside from gender, other demographic and socioeconomic variables and health-related variables were associated with health insurance enrollment. This study revealed that participants who were aged 55–64 years were 2.38 times significantly more likely to enrol in a health insurance scheme compared to those who were between 18 and 24 years (AOR = 2.38, 95%CI: 1.29–4.41, *p*-value = 0.006). This study revealed that participants affiliated with African traditional religion were 0.30 times significantly less likely to enrol in a health insurance scheme than those who were Christians (AOR: 0.30, 95%CI: 0.09–0.99, *p*-value = 0.048). This study further found that participants with tertiary education were 3.62 times significantly more likely to enrol in a health insurance scheme than those without formal education (AOR: 3.62, 95% CI: 2.32–5.66, *p*-value = 0.001). Our study has further shown that participants living with the care recipients were 1.5 times significantly more likely to enrol in a health insurance scheme than those who did not (AOR: 1.50, 95% CI: 1.14–1.98, *p*-value = 0.003). This study has also demonstrated that participants who earned between GH¢1000 and 1999 in a month were 0.7 times significantly less likely to enrol in a health insurance scheme compared to those who earned less than GH¢1000 (AOR: 0.70, 95% CI: 0.52–0.95, *p*-value = 0.022) (see Table 4).

We observed the following in Model 3 when we included a health-related variable. First, we observed that the odds of health insurance enrollment among participants aged 55–64 years increased from 2.16 in Model 2 to 2.38 in Model 3, suggesting a 22% increased in the likelihood of enrollment in health insurance schemes among this group. This suggests that a health-related variable strengthens the significant association between age (for those aged 55–64 years) and health insurance enrollment. Second, we observed that the likelihood of health insurance enrollment among participants affiliated with African traditional religion remained unchanged in Models 2 and 3 after including a health-related variable. Third, the likelihood of health insurance enrollment decreased from 3.72 in Model 2 to 3.62 in Model 3 for participants with a tertiary level of education when a health-related variable was added to demographic and socio-economic variables. This suggests that adding a health-related variable to demographic and socio-economic variables weakens the significant association between education level (specifically for those with a tertiary level) and health insurance enrollment, but the association was still significant. Fourth, the odds of health insurance enrollment reduced from 1.57 in Model 2 to 1.50 in Model 3 among participants living with their care recipients, though the association was still significant. This indicates that including a health-related variable to demographic and socio-economic variables weakens the association between living arrangements (particularly those who reside with their care recipients) and health insurance enrollment but does not render it insignificant. Last, we noted that the odds of health insurance enrollment among participants who earned between GH¢1000–1999 in a month remained unchanged in Models 2 and 3 after including a health-related variable.

## Discussion

This study, to the best of our knowledge, is the first to examine the association between gender and health insurance enrollment among informal caregivers of older adults in Ghana. The findings of this study can help inform policy to promote healthcare financing among informal caregivers of older adults in Ghana and elsewhere. Getting literature on health insurance enrollment among informal caregivers of older adults is challenging. Given this, the findings of this study have been linked to the general literature on determinants of health insurance enrollment among the general population, older adults, and persons with disabilities.

Our study revealed that the rate of health insurance enrollment was 81.1% for female participants and 64.3% for male participants. The rates of health insurance schemes among male and female participants are higher than those reported in a nationally representative survey among the general population [7, 8]. For instance, Ayanore et al. [7], drawing data from the 2014 Ghana Demographic Health Survey, found that 66% of females and 52.6% of males are enrolled in the NHIS. In their study on the determinants of health insurance enrollment from three national household surveys in Ghana, Salari et al. [8] reported that, based on the Ghana Living Standards Survey (GLSS 2012–2013), 38.1% of female and 34.4% of male have a valid health insurance card. The disparities in the findings may be due to variations in geographical coverage, unit of analysis and socio-demographics. The higher health insurance enrollment rate of male and female participants in this study could be linked to the nature of the caregiving activities, which mostly predispose them (informal caregivers) to health challenges and the need to prepare for future health problems.

This study reported an association between gender and health insurance enrollment among the participants. Specifically, the findings of this study have shown that female participants were more likely to enrol in a health insurance scheme than their male counterparts. This finding supports our key hypothesis that female informal caregivers of older adults are significantly more likely to enrol in health insurance schemes. Although not a related study, this finding is similar to a previous study that reported that compared with males with disabilities, females with disabilities have a higher likelihood of enrollment in a health insurance scheme [23]. Another Ghanaian study reported that females are more likely to enrol in health insurance schemes than males [8]. A study has further reported lower enrollment in health insurance schemes among Ghanaian males [28]. Higher likelihood of health insurance enrollment among females could be attributed to females perceiving their health status as poor compared to males. Relatedly, poor self-rated health is also associated with increased healthcare utilization [19] and more specifically, females tend to use more healthcare services than males [25]. Hence, enrollment in a health insurance scheme serves as a safety net against higher healthcare expenditure [23] associated with increased healthcare utilization, specifically among females [29]. Also, compared to males, females tend to have more access to community resources and support networks [30], which help increase their ability to afford the cost associated with enrollment in health insurance schemes. Again, females have a positive attitude towards health insurance decisions, which increases their likelihood of enrolling in a health insurance scheme [31]. Further, given that the average age of the participants was approximately 39 years, most female participants were within the reproductive age group. As such, they would enjoy a free maternal health policy under the NHIS. As a result, most female participants might have enrolled in the health insurance scheme under this exemption category [25]. However, we caution that our study did not find out from the female participants if they enjoy free enrollment in the health insurance scheme. At the same time, we did not find out whether the female participants were pregnant to enjoy free maternal healthcare.

Although not the main focus of this study, we found that other control variables were associated with health insurance enrollment among the participants. For instance, this study found that participants aged 55–64 were significantly more likely to enrol in a health insurance scheme than those aged 18–24 years. This finding supports earlier studies that have established a relationship between age and health insurance enrollment [32, 33]. This is linked to the fact that as the population ages, their health status worsens, requiring them to seek appropriate health interventions, including enrollment in a health insurance scheme [32]. Also, health needs increase as individuals age which predispose them to enrol in a health insurance scheme.

This study revealed an association between religion and health insurance enrollment. More specifically, we found that participants affiliated with African traditional religions were less likely to enrol in a health insurance scheme. In a study among informal sector workers in the Kumasi Metropolis of Ghana, Adei et al. [24] revealed that non-Christians were less likely to enroll in a health insurance scheme. We attributed this finding to disparities in religious, cultural, and healthcare-seeking behaviours [34].

Our analysis has shown that participants with tertiary education have higher odds of enrolling in a health insurance scheme than those without formal education. This finding is consistent with previous studies [5, 35]. A greater level of education gives individuals a better awareness and information of the possible physical, economic, and social consequences of ailments and the value of health insurance as safety nets in catastrophic health situations. Consequently, they are anticipated to make every conceivable effort to improve their health and well-being by enrolling in health insurance schemes [36].

Our findings revealed an association between income and health insurance enrollment among the participants. Specifically, we found that participants who earned GH¢1000–1999 in a month were less likely to enrol in a health insurance scheme than those who earned less than GH¢1000. There are mixed findings regarding the association between income and health insurance enrollment. For instance, previous Ghanaian studies have found higher wealth quintiles to be associated with higher likelihood of enrolment in health insurance schemes and lower wealth quintilesassociated with lower odds of enrollment [7, 8, 37]. Thus far, these studies contradict our current findings, which could be attributed to disparities in methods and geographical location. However, in line with our findings, Wiredu et al. [23], in their study on the prevalence of health insurance enrollment and associated factors among persons with disabilities in Ghana, found that participants with lower income were more likely to enrol in a health insurance scheme. Higher-income individuals are less likely to enrol in a health insurance scheme because of their perceived ability to afford complementary health insurance and the cost of health services, including out-of-pocket payments [23, 38].

This study has established an association between living arrangements and health insurance enrollment among the participants. More specifically, this study revealed that participants living with their care recipients were more likely to enrol in a health insurance scheme than those who did not reside with their care recipients. Higher odds of enrollment in a health insurance scheme among participants residing with their care recipients could be linked to their proximity to the care recipients. However, the above-highlighted reason may not be exhaustive, so more qualitative studies are needed to improve our understanding of the underlying reasons for higher likelihood of enrollment in a health insurance scheme among participants residing with their care recipients.

### Implications for knowledge, policy, practice and future research

Given the findings of this study, several implications for knowledge, policy and practice need to be acknowledged. In the context of the contributions to knowledge, this study provides baseline empirical and methodological literature on enrollment in a health insurance scheme among informal caregivers of older adults in Ghana. Empirically, this study contributes to the literature by highlighting variations in enrollment in health insurance schemes between male and female informal caregivers of older adults. Regarding the methodological contributions, applying the cluster sampling approach where the study area was divided into three geographical areas (Southern, Middle, and Northern) along with the large sample size enhanced the robustness of the overall methods used in this study. The methodology in this study could serve as a baseline methodological framework to guide future research on health insurance enrollment among informal caregivers of older adults in Ghana and other geographical settings with similar characteristics.

In terms of policy, the findings of this study could initiate policy discussion on gender differences in health insurance enrollment among informal caregivers of older adults in Ghana. The findings of this study are further helpful for health providers (nurses, physicians, etc.), families, care recipients and researchers, among others, to understand the association between gender and health insurance enrollment among informal caregivers of older adults. Such an understanding could help streamline the development of gender-specific health policies and programs to improve health insurance enrollment among informal caregivers of older adults.

In terms of practice, our finding that females are more likely to enrol in a health insurance scheme suggests the need to organize education and training programs for male informal caregivers of older adults to improve their enrollment in a health insurance scheme. For instance, such programs could be organized by social welfare institutions, non-governmental organizations, health providers, and the media in the various 261 districts in Ghana to elicit the views of both male and female informal caregivers of older adults on strategies to improve health insurance enrollment. As part of the education, male informal caregivers of older adults should also be encouraged to enrol in a health insurance scheme through the financial support of their families and other benevolent organizations.

### Strengths, limitations and future research

The main strength of this study is that it is the first to examine the association between gender and health insurance schemes among informal caregivers of older adults in Ghana. However, some limitations need to be acknowledged. First, due to this study’s cross-sectional nature, we could not draw any causal explanations between the outcome and the predictor variable(s). Second, this study recruited participants from one region, suggesting that results may not represent the views of all informal caregivers of older adults in Ghana. Last, we used snowball sampling technique to recruit informal caregivers of older adults, so this may limit the generalization of our results.

Due to the limitation associated with cross-sectional study, this study recommends that future works employ longitudinal data (with mixed methods design) to analyze the association between gender and health insurance enrollment among informal caregivers of older adults. Given our findings, additional qualitative studies on (1) why female older adults have higher enrollment in a health insurance scheme and (2) factors impacting health insurance enrollment among male informal caregivers of older adults should be explored. These future studies could be extended to other regions of Ghana to enhance the generalization and representativeness of the findings.

## Conclusion

This study examines the association between gender and health insurance enrollment among informal caregivers of older adults in Ghana. The findings of this study showed that the enrolment rate in health insurance schemes was high among informal caregivers of older adults. The findings specifically highlight that female informal caregivers of older adults were more likely to enrol in health insurance schemes than their male counterparts, suggesting gender gaps in health insurance enrollment. Apart from gender, other covariates such as age, religion, education, income and living arrangements with the care recipients were significantly associated with health insurance enrollment among informal caregivers of older adults. These findings suggest the need to develop gender-specific measures to improve health insurance enrollment among informal caregivers of older adults in Ghana.

## Data Availability

The datasets used and analyzed during the current study are available from the corresponding author upon reasonable request.

## Abbreviations

AOR: Adjusted Odds Ratio
CHRPE: Committee on Human Research Publication and Ethics
COR: Crude Odds Ratio
GREB: General Research Ethics Board
NHIS: National Health Insurance Scheme
SDGs: Sustainable Development Goals
UHC: Universal Health Coverage

## References

[1] UN Department of Economic and Social Affairs (n.d). Transforming our world: the 2030 Agenda for sustainable development. Accessed from, https://sdgs.un.org/2030agenda

[2] Hsiao WC, Shaw RP (2007). Lessons learned and policy implications. Social Health Insurance Developing Nations.

[3] Mills A, Ataguba JE, Akazili J, Borghi J, Garshong B, Makawia S, McIntyre D (2012). Equity in financing and use of health care in Ghana, South Africa, and Tanzania: implications for paths to universal coverage. Lancet.

[4] Christmals CD, Aidam K. (2020). Implementation of the National health insurance scheme (NHIS) in Ghana: lessons for South Africa and low-and middle-income countries. Risk Manage Health care Policy, 1879–904. 10.2147/RMHP.S245615 PMC7537808 33061721

[5] National Health Insurance Authority, Ghana. (2023). Membership. National Health Insurance Scheme. Accessed at, Membership (nhis.gov.gh).

[6] Ministry of Health, Ghana (2022). Health sector annual programme of work: 2021 holistic Assessment Report.

[7] Ayanore MA, Pavlova M, Kugbey N, Fusheini A, Tetteh J, Ayanore AA, Groot W (2019). Health insurance coverage, type of payment for health insurance, and reasons for not being insured under the National Health Insurance Scheme in Ghana. Health Econ Rev.

[8] Salari P, Akweongo P, Aikins M, Tediosi F (2019). Determinants of health insurance enrollment in Ghana: evidence from three national household surveys. Health Policy Plann.

[9] Ayitey A, Nketiah-Amponsah E, Barimah A (2013). Determinants of insurance enrollment among Ghanaian adults: the case of the National Health Insurance Scheme (NHIS). Econ Manage Financial Markets.

[10] Dixon J, Tenkorang EY, Luginaah I (2011). Ghana’s National Health Insurance Scheme: helping the poor or leaving them behind?. Environ Plann C Gov Policy.

[11] Seddoh A, Sataru F (2018). Mundane? Demographic characteristics as predictors of enrollment onto the National Health Insurance Scheme in two districts of Ghana. BMC Health Serv Res.

[12] Van der Wielen N, Channon AA, Falkingham J (2018). Universal health coverage in the context of population ageing: what determines health insurance enrollment in rural Ghana?. BMC Public Health.

[13] Blanchet NJ, Fink G, Osei-Akoto I (2012). The effect of Ghana’s National Health Insurance Scheme on health care utilization. Ghana Med J.

[14] Gobah FF, Liang Z (2011). The National Health Insurance Scheme in Ghana: prospects and challenges: a cross-sectional evidence. Global J Health Sci.

[15] Dixon J, Luginaah I, Mkandawire P (2014). The National Health Insurance Scheme in Ghana’s Upper West Region: a gendered perspective of insurance acquisition in a resource-poor setting. Soc Sci Med.

[16] Alatinga KA, Williams JJ (2015). Towards universal health coverage: exploring the determinants of household enrollment into National Health Insurance in the Kassena Nankana District, Ghana. Ghana J Dev Stud.

[17] Peiman H, Yaghoubi M, Mohammadi S, Delpishe A (2012). Prevalence of chronic diseases in the elderly in Ilam. Iran J Ageing.

[18] Habibi A, Nemadi VM, Habibi S, Mohammadi M (2012). Quality of life and prevalence of chronic illnesses among elderly people: a cross-sectional survey. J Health Hygiene.

[19] Agyemang-Duah W, Rosenberg MW (2023). Health care utilization among informal caregivers of older adults in the Ashanti region of Ghana: a study based on the health belief model. Archives Public Health.

[20] Agyemang-Duah W, Abdullah A, Rosenberg MW (2024). Caregiver burden and health-related quality of life: a study of informal caregivers of older adults in Ghana. J Health Popul Nutr.

[21] Kumar R, Kaur S, Reddemma K (2015). Burden and coping strategies in caregivers of stroke survivors. J Neurol Neurosci.

[22] Penning MJ, Wu Z (2016). Caregiver stress and mental health: impact of caregiving relationship and gender. Gerontologist.

[23] Wiredu DNA, Peprah C, Agyemang-Duah W (2021). Prevalence of health insurance enrollment and associated factors among persons with disabilities in Ghana. Cogent Med.

[24] Adei D, Agyemang-Duah W, Mensah AA (2019). Predictors of enrollment in a health protection scheme among informal sector workers in Kumasi Metropolis of Ghana. BMC Res Notes.

[25] Duku SKO (2018). Differences in the determinants of health insurance enrollment among working-age adults in two regions in Ghana. BMC Health Serv Res.

[26] Breiman L (2001). Random forests. Mach Learn.

[27] James G, Witten D, Hastie T, Tibshirani R (2013). An introduction to statistical learning.

[28] Nsiah-Boateng E, Nonvignon J, Aryeetey GC, Salari P, Tediosi F, Akweongo P, Aikins M (2019). Sociodemographic determinants of health insurance enrollment and dropout in urban district of Ghana: a cross-sectional study. Health Econ Rev.

[29] Patel R, Chauhan S (2020). Gender differential in health care utilization in India. Clin Epidemiol Global Health.

[30] Hajek A, Gyasi RM, König HH. (2024). Factors associated with loneliness among individuals aged 80 years and over: findings derived from the nationally representative old age in Germany (D80+) study. Arch Gerontol Geriatr, 105443. 10.1016/j.archger.2024.105443 38631279

[31] Boateng D, Awunyor-Vitor D (2013). Health insurance in Ghana: evaluation of policy holders’ perceptions and factors influencing policy renewal in the Volta region. Int J Equity Health.

[32] Mulenga J, Mulenga MC, Musonda KM, Phiri C (2021). Examining gender differentials and determinants of private health insurance coverage in Zambia. BMC Health Serv Res.

[33] Amu H, Dickson KS, Kumi-Kyereme A, Darteh EKM. (2018). Understanding variations in health insurance coverage in Ghana, Kenya, Nigeria, and Tanzania: evidence from demographic and health surveys. PLoS ONE, 13(8), e0201833. 10.1371/journal.pone.0201833 PMC6078306 30080875

[34] Badu E, Agyei-Baffour P, Ofori Acheampong I, Preprah Opoku M, Addai-Donkor K (2018). Households sociodemographic profile as predictors of health insurance uptake and service utilization: a cross-sectional study in a municipality of Ghana. Adv Public Health.

[35] Jehu-Appiah C, Aryeetey G, Spaan E, De Hoop T, Agyepong I, Baltussen R (2011). Equity aspects of the National Health Insurance Scheme in Ghana: who is enrolling, who is not and why?. Soc Sci Med.

[36] Akazili, J., Welaga, P., Bawah, A., Achana, F. S., Oduro, A., Awoonor-Williams, J.K., … Phillips, J. F. (2014). Is Ghana’s pro-poor health insurance scheme really for the poor? Evidence from Northern Ghana. *BMC health services research*, *14*, 1–9. 10.1186/s12913-014-0637-7 PMC4268792 25494816

[37] Adjei-Mantey K, Horioka CY (2023). Determinants of health insurance enrollment and health expenditure in Ghana: an empirical analysis. Rev Econ Househ.

[38] Akazili J. (2010). Equity in Health Care Financing in Ghana. Doctoral dissertation, University of Cape Town, Cape Town, South Africa.

